# Phosphate induces inflammation and exacerbates injury from cigarette smoke in the bronchial epithelium

**DOI:** 10.1038/s41598-023-32053-1

**Published:** 2023-03-25

**Authors:** Seth Bollenbecker, Kylie Heitman, Brian Czaya, Molly Easter, Meghan June Hirsch, Shia Vang, Elex Harris, E. Scott Helton, Jarrod W. Barnes, Christian Faul, Stefanie Krick

**Affiliations:** 1 Division of Pulmonary, Allergy and Critical Care Medicine, Department of Medicine, The University of Alabama at Birmingham, 1918 University Blvd, MCLM 718, Birmingham, AL 35294 USA; 2 Section of Mineral Metabolism, Division of Nephrology, Department of Medicine, The University of Alabama at Birmingham, Birmingham, AL USA; 3 Department of Medicine, David Geffen School of Medicine at UCLA, Los Angeles, CA USA

**Keywords:** Cell signalling, Cell biology, Nephrology, Chronic kidney disease, Respiratory tract diseases, Chronic obstructive pulmonary disease

## Abstract

An elevation in serum phosphate—also called hyperphosphatemia—is associated with reduced kidney function in chronic kidney disease (CKD). Reports show CKD patients are more likely to develop lung disease and have poorer kidney function that positively correlates with pulmonary obstruction. However, the underlying mechanisms are not well understood. Here, we report that two murine models of CKD, which both exhibit increased serum levels of phosphate and fibroblast growth factor (FGF) 23, a regulator of phosphate homeostasis, develop concomitant airway inflammation. Our in vitro studies point towards a similar increase of phosphate-induced inflammatory markers in human bronchial epithelial cells. FGF23 stimulation alone does not induce a proinflammatory response in the non-COPD bronchial epithelium and phosphate does not cause endogenous FGF23 release. Upregulation of the phosphate-induced proinflammatory cytokines is accompanied by activation of the extracellular-signal regulated kinase (ERK) pathway. Moreover, the addition of cigarette smoke extract (CSE) during phosphate treatments exacerbates inflammation as well as ERK activation, whereas co-treatment with FGF23 attenuates both the phosphate as well as the combined phosphate- and CS-induced inflammatory response, independent of ERK activation. Together, these data demonstrate a novel pathway that potentially explains pathological kidney-lung crosstalk with phosphate as a key mediator.

## Introduction

Chronic kidney disease (CKD) is characterized by renal damage and a loss of functional nephrons that persists for at least three months^1,2^. Among the many consequences of CKD, damaged kidneys are unable to remove excess phosphate from the blood as effectively, resulting in elevated serum phosphate levels^3^. This hyperphosphatemia triggers elevations in fibroblast growth factor (FGF) 23 levels, a bone-derived hormone responsible for suppressing renal phosphate reabsorption^4^. Disruptions in this mineral homeostasis have been linked to cardiovascular^5^ and bone disease^6^, hyperparathyroidism^7^, and anemia^8^, as well as renal failure from which these diseases can originate. However, there is limited research surrounding the consequences of CKD on lung function. Individuals with renal failure are more likely to develop pulmonary complications^9,10^ and the number of accompanying comorbidities increases as kidney function declines^11^, leaving a gap of knowledge that our study aims to fill.

Prevalence of obstructive lung dysfunction, a classification encompassing diseases such as chronic obstructive pulmonary disease (COPD), increases four-fold (from 9 to 36%) over the course of CKD progression from stage 1 to stage 5^10,11^. COPD is the third leading cause of death worldwide, with an estimated 328 million cases globally^12^. Smoking is the primary cause of COPD^13^ and significantly increases the risk of developing CKD and exacerbating existing renal failure^9,14^, establishing it as a key player in CKD and COPD-associated kidney-lung crosstalk. Smoking has also been found to correlate with higher levels of serum phosphate^15^, further complicating matters for CKD patients with existing hyperphosphatemia and elevated FGF23 levels. Although we have previously shown that plasma FGF23 is increased in patients with mild COPD and that FGF23 can exacerbate cigarette smoke (CS)-induced inflammation in bronchial epithelial cells from COPD patients^16^, studies examining the effects of dysregulated phosphate homeostasis on lung cells are lacking.

Pathological changes in COPD include airway wall thickening, goblet cell hyperplasia, and an increased fractional volume of bronchial epithelial cells^17,18^. Bronchial epithelial cells are particularly vulnerable to the effects of CS, as they are some of the first cells exposed to external particulate matter and irritants^19^. Previous studies have shown that exposure to CS activates the mitogen-activated protein kinase (MAPK)/extracellular signal-regulated kinase 1/2 (ERK) signaling network^20,21^, leading to increased production of proinflammatory cytokines such as IL(interleukin)1B, IL6, and IL8. Given that this same signaling pathway has been identified as a key player in phosphate and FGF23-mediated inflammation in the kidney^2^, parathyroid glands^22^, and in vascular smooth muscle cells^23^, it is of particular interest to our exploration of kidney-lung crosstalk.

Therefore, the aims of this study were twofold: (1) establish whether disruptions in phosphate homeostasis have an impact on bronchial epithelial cells and (2) determine if these imbalances play a role in increasing susceptibility to CS-induced inflammation in the bronchial epithelium. Previous findings show that hyperphosphatemia and/or increases in FGF23 induce inflammation in the kidney^2^, liver^24^, heart^25^, and parathyroid glands^22^. This mineral and hormonal imbalance may be a culprit of CKD-associated lung disease susceptibility. Therefore, we hypothesize that increased phosphate and FGF23 levels contribute to inflammation in bronchial epithelial cells and exacerbate existing inflammation induced by CS.

## Materials and methods

### Mice

Animal studies were performed in conformity with applicable laws and guidelines as well as being approved by the Animal Research Committee at the University of Alabama at Birmingham (UAB) School of Medicine. Mice were housed at UAB in accordance with federal and university guidelines for the care and use of experimental vertebrate animals and protocols were reviewed and approved by the UAB Institutional Animal care and Use Committee (IACUC). Authors have complied with the ARRIVE guidelines for reporting. Studies were performed using both male and female mice that were maintained on a NIH 31 rodent diet (Harlan Teklad) and fed ad libitum, unless otherwise indicated. Constitutive *Col4a3*^−/−^ null (Alport) mice were maintained on a mixed Sv129/C57BL/6 background^26^.

For experiments involving adenine-induced kidney failure, a dietary timeline previously described was used^8,27^. Briefly, 10- to 14-week-old mice on a C57BL/6 background were placed on a customized diet containing 0.2% adenine (TD.140290, Envigo) for 6 weeks, switched to a customized diet containing 0.15% adenine (TD.170304, Envigo) for 2 weeks, then transitioned back to the 0.2% adenine diet for 6 more weeks. Littermates placed on a control diet (TD.170303, Envigo) were used for controls. After a 1-week diet acclimation period, mice were randomly assigned to the control or adenine diet group. Following the 14-week dietary period, mice were euthanized under 2.5% isoflurane anesthesia and samples were prepared as described below. Excess adenine causes formation of insoluble 2,8-dihydroxyadenine crystals, which induce kidney tubule-interstitial damage and is commonly used to study CKD^28^.

For experiments focused on phosphate and FGF23 dysregulation in CKD-associated pathologies, *Col4a3*^−/−^ mice and corresponding wild-type littermates were euthanized at 10 weeks of age under 2.5% isoflurane anesthesia and samples were prepared as described below. Constitutive *Col4a3*^−/−^ null mice are considered a genetic model of human Alport syndrome and progressive CKD^26^. These mice develop proteinuria and hematuria due to glomerulonephritis, consistent with CKD, which results from the absence of collagen type IV in the glomerular basement membrane of the kidney^26,29^. Mice maintained on a Sv129/C57BL/6 mixed background die around 10 weeks of age due to renal injury.

### Serum chemistry

Murine blood was collected by cardiac puncture, dispensed into microvette serum tubes (365967, Becton, Dickinson and Company), and allowed to rest undisturbed for 30 min. Samples were then centrifuged at 10,000×*g* for 5 min at room temperature. Serum supernatants were separated and stored at − 80 °C. Serum intact FGF23 (biologically active form, before cleavage into C- and N-Terminal fragments^30^) was assessed using a commercially available ELISA kit (60-6800, Quidel). Clinical chemistry analyses including blood urea nitrogen (BUN), creatinine, and serum phosphate concentration were performed by IDEXX BioAnalytics (6006-UC, comprehensive panel).

### Bronchoalveolar lavage (BAL) and cell quantification from BAL fluid in mice

Bronchoalveolar lavage fluid (BALF) was obtained following protocols previously established in our lab^16^. In brief, mice were euthanized and a cannula was inserted into a small hole in the trachea. The cannula was then tied off to prevent fluid leaks. Cold PBS (1 mL) was used to lavage the lungs under direct visualization of lung distension and withdrawn. Cells were pelleted by centrifugation and resuspended in 9 parts sterile water for 30 s to allow for lysis of red blood cells, followed by the addition of 1-part 10 × PBS to bring the final isotonic concentration to 1 × PBS. Charged microscope slides (12-550-15, Fisher Scientific) were centrifuged at 300×*g* for 5 min (CytoSpin 4, Thermo Scientific). Each slide was stained with Hema 3 (123-869, Fisher Scientific) and macrophages, lymphocytes, and neutrophils were quantified using randomly selected fields until a total cell count of 400 was reached.

### Lung histology and immunohistochemical staining

Mice were euthanized and the lungs were perfused via the right ventricle with 3 mL of PBS. Using a catheter (26751, Exel International) inserted into the trachea, the lungs were inflated with 1 mL of 10% formalin solution, removed, and subsequently submerged in 10% formalin solution at room temperature for 24 h. The lungs were then transferred into 70% ethanol and subjected to paraffin embedding, sectioning, and hematoxylin and eosin (H&E), Alcian blue periodic acid-Schiff (ABPAS), or trichrome staining by the Comparative Pathology Laboratory at UAB. Lung sections were also subjected to immunohistochemical (IHC) staining with IL1B (AF-401-NA, R&D Systems) or CXCL2 (701126, Thermo Scientific) using a mouse and rabbit HRP/DAB detection kit (ab64264, abcam). Images were captured on a Nikon Ts2-FL inverted microscope with a Digital Sight 10 camera or a Keyence BZ-X800 digital microscope.

For quantification of bronchial epithelial thickness and goblet cell size, randomly selected regions of each ABPAS-stained slide were chosen for measurement and analyzed as previously described^31,32^. Briefly, bronchial epithelial cells were measured from their base to their highest point in the lumen. Goblet cell size was quantified by calculating the area of randomly selected goblet cells and normalized to the luminal area of the corresponding airway from each animal.

### Cell culture

Human bronchial epithelial cells (BEAS-2B, CRL-9609, ATCC) were cultured in Bronchial Epithelial Cell Growth Basal Medium (CC-3171, Lonza) with Bronchial Epithelial Cell Growth Medium SingleQuots Supplements and Growth Factors (CC-4175, Lonza) on tissue culture treated plates coated with collagen type IV (C7521, Sigma-Aldrich) as previously described^16^. Cells were treated with appropriate amounts of sodium phosphate (0.5 M; pH 7.4) and sodium sulfate (0.5 M; pH 7.4) buffers, prepared as previously described^8^, to produce final desired concentrations and incubated 24 h in a humidified 5% CO_2_ incubator at 37 °C. Sodium sulfate served as a negative control in response to increased anion balance. In experiments where inhibitors were used, cells were pretreated with a fibroblast growth factor receptor (FGFR)1–3 inhibitor (AZD4547), FGFR4 inhibitor (BLU-554), phosphonoformic acid (PFA, P6801, Sigma) as a sodium-dependent phosphate cotransporter inhibitor^33^, or mitrogen-activated protein kinase kinase (MEK) inhibitor (U0126), used to inhibit phosphorylation of extracellular signal regulated kinase (ERK), for one hour before the addition of phosphate. Viability of cells post-treatment was assessed using a trypan blue exclusion test of cell viability as previously described^34^.

CSE was prepared using a modification of a previously described method^16,35^ to increase reproducibility amongst experiments. Briefly, this was done by bubbling cigarette smoke through 1 mL basal medium, per cigarette (3R4F, University of Kentucky), followed by passage through a 0.45 µM filter for sterilization. For experimental purposes, this stock CSE solution was applied to BEAS-2B cell cultures in a dose-dependent manner for 24 h and mRNA transcript levels of *IL1B* were analyzed to establish a dose–response curve. A two-fold increase in gene expression of *IL1B* was used as the target CSE treatment dose and all experiments utilizing CSE were diluted from the stock solution in this manner.

### ELISA

IL6 and IL8 cytokine concentrations from conditioned cell culture media were measured using a human IL6 (88-7066-88, Invitrogen) and a human IL8 (88-8086-86, Invitrogen) enzyme-linked immunosorbent assay (ELISA). Media from BEAS-2B cell cultures was collected after treatment at indicated time points, clarified by centrifugation at 500×*g* for 10 min at 4 °C, loaded onto an assay plate coated with anti-IL6 or anti-IL8 capture antibody, and incubated on a plate shaker at 220 RPM for 2 h at room temperature. After completing the manufacturer’s included protocol, absorbance values were measured at 450 nm and concentrations of each cytokine were determined using suggested dilutions of an included standard.

### RNA purification and Quantitative Real Time PCR (qRT-PCR)

Total RNA was extracted from mouse lung tissue using the Mini Total RNA Kit (IB47302, IBI Scientific) and from BEAS-2B cell cultures using the GeneJET RNA purification kit (K0732, Thermo Scientific). Employing a two-step reaction method, 1 µg of total RNA was reverse transcribed into cDNA using Maxima H Minus cDNA Synthesis Master Mix (M1669, Thermo Scientific) in a thermocycler (PCT-100, MJ Research) with the manufacturer’s suggested protocol. For gene expression analysis, qRT-PCR was performed with 25 ng of cDNA using the following Taqman probes (Life Technologies/Applied Biosystems): Mm00434228_m1 for mouse *Il1b*, Mm00436450_m1 for mouse Cxcl2/Mip2, Hs01555410_m1 for human *IL-1B*, Hs00174131 for human *IL6*, Hs00174103_m1 for human *IL8*, Hs00241111_m1 for human *FGFR1*, and Hs01106910_g1 for human *FGFR4*. Our internal control used for normalization of gene expression values was Hs02758991 for GAPDH. All qRT-PCR was carried out on a StepOnePlus Real-Time PCR System (Applied Biosystems) and results were evaluated using the 2^−∆∆CT^ method.

### Western immunoblotting

Cell lysates were prepared in RIPA (radioimmunoprecipitation assay) buffer (9806, Cell Signaling) with Halt protease and phosphatase inhibitor cocktail (78430, Thermo Scientific). Protein yield was measured by Bradford protein assay (1856210, Pierce). Proteins (40 µg total protein) were separated on a 4–20% precast Ready Gel (Bio-Rad) and blotted onto nitrocellulose blotting membranes with 0.45 µM pores (10600003, Amersham). Membranes were then blocked with 5% BSA for phosphorylated proteins or 5% nonfat milk (for βactin) in Tris-buffered saline (pH 7.4) with 0.05% Tween-20 (TBST) for 30 min. Membranes were probed for primary antibodies at 1:1000 against specific antigens overnight at 4 °C. ERK1/2 (4695, Cell Signaling), pERK1/2 (9101, Cell Signaling), AKT (9272, Cell Signaling), and pAKT at Ser473 (4060, Cell Signaling) antibodies were used in TBST.

Next day, membranes were washed and probed with horseradish peroxidase (HRP)-conjugated goat anti-mouse (31430, Invitrogen) or goat anti-rabbit (31466, Invitrogen) secondary antibodies at 1:6000 in TBST at room temperature for 1 h. Membranes were then washed again and HRP activity was detected using enhanced chemiluminescence detection solution (1705060, Bio-Rad) and imaged on a Bio-Rad Gel Doc XR Gel Documentation System. All immunoblots were repeated for at least three independent trials with comparable results.

### Measurement of H_2_O_2_ release

In vitro H_2_O_2_ concentrations were measured using a previously described fluorimetric method based on the conversion of homovanillic acid to its dimerized form in the presence of H_2_O_2_ and horseradish peroxidase^36^. Briefly, after exposure to each treatment for 24 h, cells were washed with Hank’s balanced salt solution (HBSS, H8264, Sigma) and then incubated with a reaction mixture composed of 100 µM homovanillic acid (H1252, Sigma) and 5 units/mL horseradish peroxidase, type VI (P8375, Sigma) in HBSS. This solution was then collected following a two-hour incubation period and the pH was adjusted to 10.0 with 0.1 M glycine–NaOH buffer. Fluorescence was measured at excitation and emission wavelengths of 321 and 421, respectively, using a black-walled, clear bottom 96-well tissue culture dish (165305, Thermo Scientific). All incubations of experimental samples were made with control samples that contained only the reaction mixture, absent of any cells to correct for spontaneous dimerization of homovanillic acid. Generation of a standard curve was created using known concentrations of H_2_O_2_ added to reaction buffer measured with each plate of the treated samples.

### Statistics

All results are expressed as mean ± SEM. Student’s *t-*tests were used to analyze group differences for continuous variables. For data sets with two or more predictor variables, a one-, two-, or three-way analysis of variance (ANOVA) was used to analyze group differences, followed by Tukey’s multiple comparison post hoc test. Comparisons between the means of all groups were carried out, but graphical representation of significance was shown for those referenced in the text for visual clarity. Details on specific tests used are included in each figure legend. A p-value of less than 0.05 was considered statistically significant in data analysis. Data were organized, graphed, and analyzed with Prism version 9 (GraphPad, La Jolla, CA, USA). Sample size was determined based on sample availability, previous experimental studies performed in our laboratory, and literature published on the subject in question.

## Results

### *Col4a3*^−/−^ mice develop CKD, which is accompanied by pulmonary inflammation and thickened bronchial epithelium

**Figure 1:**
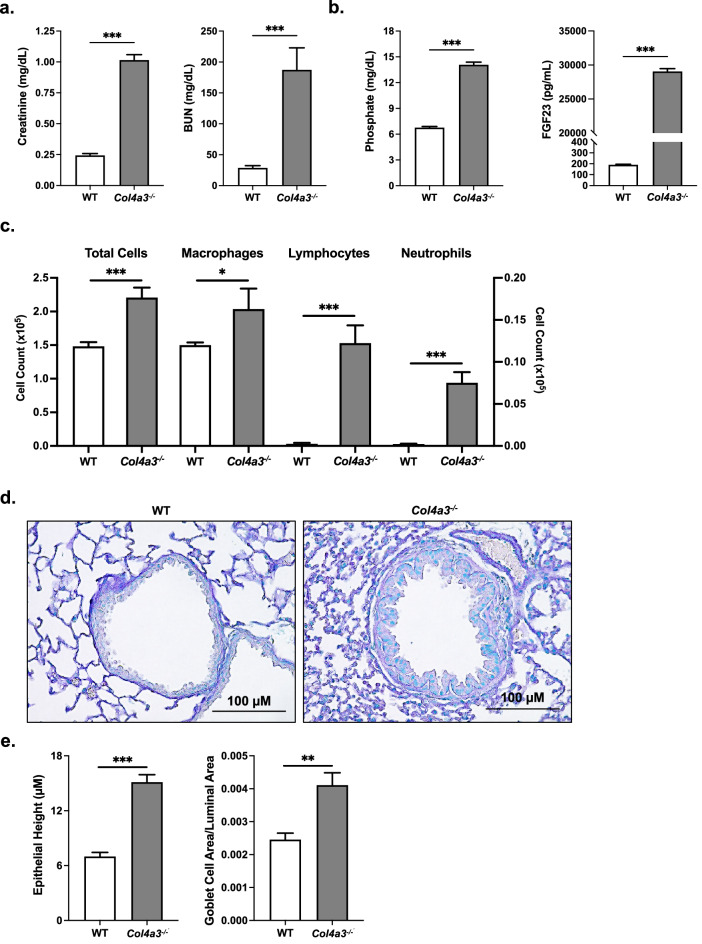
*Col4a3*^−/−^ mice have altered bronchiole histology and pulmonary inflammation. (**a**) Serum creatinine and BUN levels, (**b**) serum phosphate and FGF23 levels, and (**c**) total cell count and macrophage, lymphocyte, and neutrophil counts from BAL fluid of *Col4a3*^−/−^ mice and their wild type littermates. Left y-axis applies to total cell and macrophage count; right y-axis applies to lymphocyte and neutrophil count. (**d**) Representative pathology of ABPAS-stained lung sections (scale bar, 100 µM) from mouse lungs of both groups. (**e**) Height of epithelial layer and goblet cell area/luminal area quantified from ABPAS histology images; 3 regions quantified from each representative mouse lung. All values are mean ± SEM with *p ≤ 0.05, **p < 0.01, and ***p < 0.001 and *n* = 8 mice/group. Statistical significance was analyzed via unpaired Student’s *t*-test (**a**–**e**).

To examine whether disruptions in phosphate homeostasis as a result of kidney injury can induce notable pulmonary changes in vivo, we analyzed the lungs of two commonly used murine CKD models. For the first of these models, we used Alport (*Col4a3*^−/−^) mice, which have been characterized for renal dysfunction previously^26,37^. Compared to wild-type (WT, *Col4a3*^+/+^) mice, the *Col4a3*^−/−^ mice exhibited signs of renal dysfunction as demonstrated by significant increases in serum creatinine and BUN levels (Fig. 1a). In addition, the *Col4a3*^−/−^ mice also displayed increased levels of serum phosphate and intact FGF23 (Fig. 1b). To determine the potential effects CKD exerts on airway inflammation, we collected BALF at the time of harvest. Total cell count was increased in the BALF of the *Col4a3*^−/−^ mice as well as the total number of macrophages, lymphocytes, and neutrophils (Fig. 1c), indicating presence of airway inflammation. Lungs were stained with Alcian blue and periodic acid-Schiff (ABPAS), revealing a much thicker bronchial epithelial layer and bronchial epithelial cell hypertrophy in the *Col4a3*^−/−^ mice when compared to the WT controls (Fig. 1d). Height of the bronchial epithelium and goblet cell area in comparison to the luminal area further validated the qualitative disparities observed between the WT and *Col4a3*^−/−^ mouse lungs (Fig. 1e). In summary, *Col4a3*^−/−^ mice not only demonstrate hyperphosphatemia and excess FGF23 levels but also lung-associated airway pathology, including inflammation and remodeling.

### Mice with adenine-induced CKD exhibit an upregulation of proinflammatory cytokines in lung tissue and the bronchial epithelium

**Figure 2:**
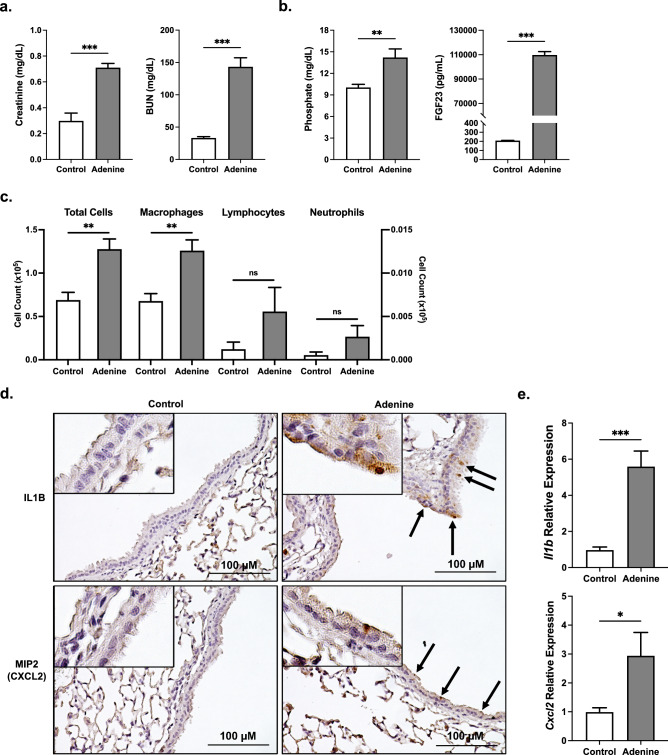
Adenine-induced CKD mice exhibit lung inflammation. (**a**) Serum creatinine and BUN levels, (**b**) serum phosphate and FGF23 levels, and (**c**) total cell count and macrophage, lymphocyte, and neutrophil counts from BAL fluid of mice on the adenine diet and littermates on the control diet. Left y-axis applies to total cell and macrophage count; right y-axis applies to lymphocyte and neutrophil count. (**d**) Representative pathology of IL1B and CXCL2 IHC-stained lung sections (scale bar, 100 µM) from mouse lungs of both groups. (**e**) Relative mRNA transcript levels of *Il1b* and *Cxcl2* in total mouse lung homogenates. All values are mean ± SEM with *p ≤ 0.05, **p < 0.01, and ***p < 0.001 and *n* = 10 mice/group. Statistical significance was analyzed via unpaired Student’s *t*-test (**a**–**e**).

Mice on an adenine-enriched diet for 14 weeks had significant increases in creatinine and BUN levels when compared to mice on a control diet (Fig. 2a). On the adenine-enriched diet, serum phosphate and FGF23 levels were also significantly elevated (Fig. 2b), consistent with renal dysfunction. Comparable to the genetic CKD mouse model (*Col4a3*^−/−^), total cell count and the number of macrophages present in the BAL fluid were significantly increased in the mice on the adenine-enriched diet, compared to those fed a control diet. No differences were seen in the lymphocyte and neutrophil counts (Fig. 2c). IHC revealed higher IL1B and MIP (macrophage inflammatory protein)-2/CXCL (C-X-C Motif Chemokine Ligand) 2 protein expression predominantly in the bronchial epithelium of the mice on an adenine-enriched diet (Fig. 2d). Furthermore, gene expression of *Il1 beta* and *Cxcl2* cytokines were also significantly increased in the total lung homogenate from the adenine diet mice (Fig. 2e). In summary, an established diet-induced CKD mouse model demonstrates airway inflammation and upregulation of proinflammatory markers in the whole lung and bronchial epithelium.

### FGF23 alone does not induce inflammation in cultured human bronchial epithelial cells

**Figure 3:**
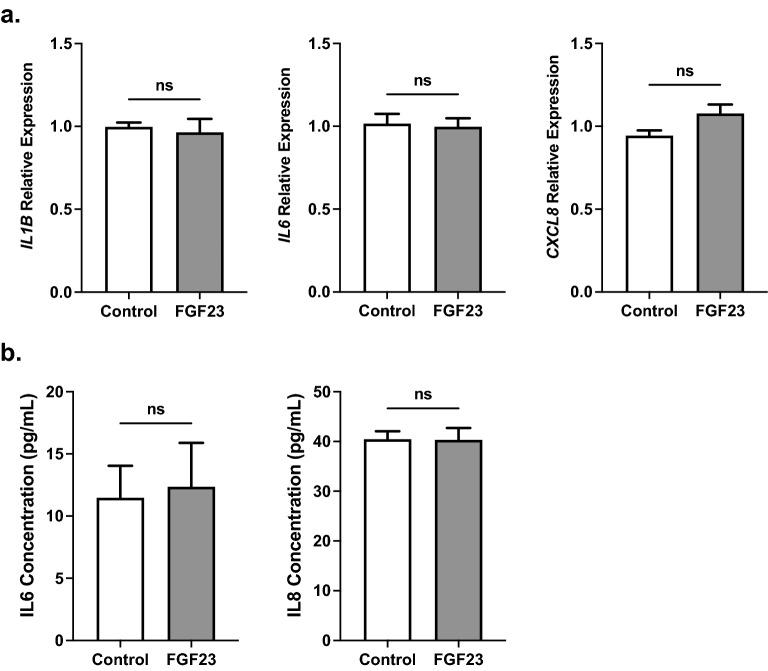
FGF23 does not induce inflammatory cytokine upregulation or secretion in HBECs. (**a**) Relative mRNA transcript levels of *IL1B*, *IL6*, and *IL8* in BEAS-2B HBECs after 24 h FGF23 stimulation. (**b**) IL6 and IL8 protein levels in conditioned media. All experiments were reproduced 3 times with triplicates. All values are mean ± SEM with *p ≤ 0.05, **p < 0.01, and ***p < 0.001. Statistical significance was analyzed via unpaired Student’s *t*-test (**a**,**b**).

Since our in vivo results suggested an inflammatory phenotype in the lungs and specifically the bronchial epithelium, we wanted to test whether FGF23 could elicit inflammatory markers in human bronchial epithelial cell (HBEC) cultures (in vitro)*.* This hypothesis was also supported by our previous findings showing that FGF23 could exaggerate CS-induced upregulation of IL1B in bronchial epithelial cells from donors with COPD^16^. BEAS-2B HBECs were treated with 40 ng/mL FGF23 as established previously^38^. Treatment had no effect on *IL1B*, *IL6*, or *CXCL8* expression levels after 24 h (Fig. 3a). Similarly, conditioned media collected after treatment with FGF23 also showed no difference in secreted IL6 or IL8 levels (Fig. 3b). IL1β levels were undetectable in the media from FGF23-treated BEAS-2B cells. These data suggest that FGF23 alone has no effect on inflammatory cytokine gene expression or secretion in BEAS-2B cells.

### Phosphate induces expression of proinflammatory cytokines and activates ERK signaling in BEAS-2B cells

**Figure 4:**
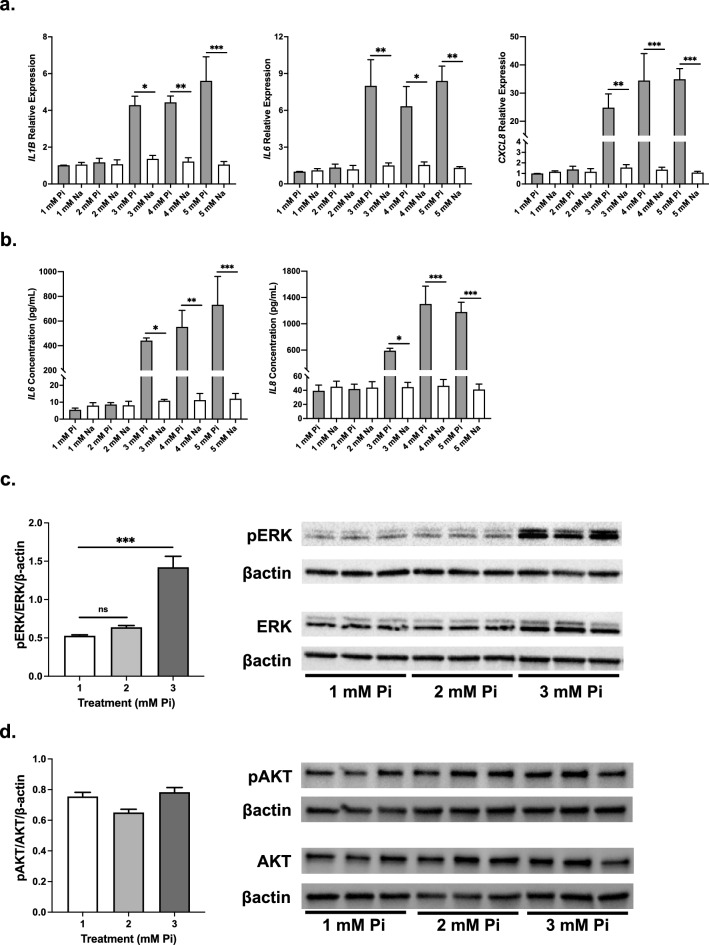
Phosphate induces inflammatory cytokine expression, secretion, and ERK activation in HBECs. (**a**) Relative mRNA transcript levels of *IL1B*, *IL6*, and *IL8* in BEAS-2B HBECs after 24 h sodium phosphate (Pi) or sodium sulfate (Na) treatment. (**b**) IL6 and IL8 protein levels in conditioned media. (**c**) Representative immunoblots and quantification of ERK phosphorylation in total protein extracts from BEAS-2B HBECs. βactin serves as loading control. (**d**) Representative immunoblots and quantification of AKT phosphorylation in total protein extracts from BEAS-2B HBECs. βactin serves as loading control. All experiments were reproduced 3 times with triplicates. All values are mean ± SEM with *p ≤ 0.05, **p < 0.01, and ***p < 0.001. Original western blots presented are available in Supplemental Fig. S1. Statistical significance was analyzed via two-way (**a**,**b**) or one-way (**c**,**d**) ANOVA followed by Tukey’s multiple comparison post hoc test.

BEAS-2B HBECs were treated with increasing concentrations of sodium phosphate (denoted simply as phosphate) or sodium sulfate. Given that physiological levels of serum phosphate are around 1 mM in healthy patients^39^, we used this as our control as well as sodium sulfate for an ion-balanced control at each concentration. Beginning at a 3 mM phosphate concentration in the cell culture media, *IL1B*, *IL6*, and *CXCL8* mRNA levels were significantly increased with respect to the physiological 1 mM phosphate control as well as their respective ion-balanced sodium sulfate controls (Fig. 4a). Similarly, IL6 and IL8 protein levels were increased in the conditioned media at 3, 4, and 5 mM phosphate compared to both the physiological 1 mM phosphate control and each respective ion-balanced sodium sulfate control (Fig. 4b). Given previous findings suggesting phosphate can induce inflammation through ERK and AKT activation in other cell types^40^, we examined phosphorylation of ERK and AKT after treating with increasing concentrations of phosphate in HBECs. Treatment with 3 mM phosphate for 24 h induced significant ERK phosphorylation (Fig. 4c). No changes were seen in the phosphorylation of AKT at any of the tested phosphate concentrations (Fig. 4d). Collectively, these data indicate that phosphate can upregulate proinflammatory cytokine expression as well as activate ERK signaling in BEAS-2Bs.

### Inhibition of ERK signaling prevents phosphate-induced upregulation of inflammatory cytokines, which is independent of FGFR signaling

**Figure 5:**
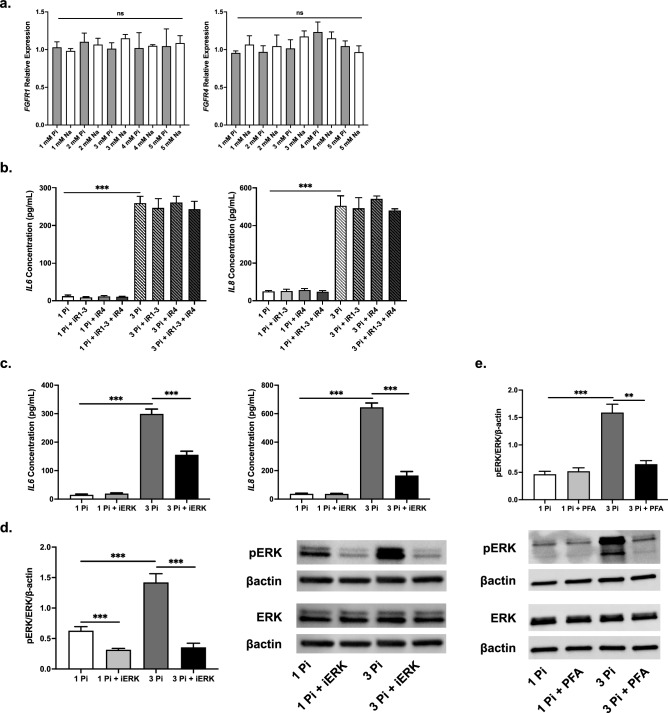
Inhibition of ERK, but not isoform-specific inhibition of FGFRs, attenuates inflammatory cytokine secretion in HBECs. (**a**) Relative mRNA transcript levels of *FGFR1* and *FGFR4* in BEAS-2B HBECs after 24 h sodium phosphate (Pi) or sodium sulfate (Na) stimulation. (**b**) IL6 and IL8 protein concentrations in conditioned media after Pi stimulation with iR1-3 and/or iR4 pretreatment. (**c**) IL6 and IL8 protein levels in conditioned media after Pi stimulation with and without iERK pretreatment. (**d**) Representative immunoblots and quantification of ERK phosphorylation in total protein extracts from BEAS-2B HBECs after Pi stimulation with and without iERK pretreatment. βactin serves as loading control. (**e**) Representative immunoblots and quantification of ERK phosphorylation in total protein extracts from BEAS-2B HBECs after Pi stimulation with and without PFA pretreatment. βactin serves as loading control. All experiments were reproduced 3 times with triplicates. All values are mean ± SEM with *p ≤ 0.05, **p < 0.01, and ***p < 0.001. Original western blots presented are available in Supplemental Fig. S2. Statistical significance was analyzed via two-way (**a**,**c**–**e**) or three-way (**b**) ANOVA followed by Tukey’s multiple comparison post hoc test.

Following up on previous findings that phosphate and FGF23 can signal through fibroblast growth factor receptor (FGFR) 1^41^ and 4^4^, respectively, we decided to examine whether these receptors play a role in the phosphate-induced upregulation of proinflammatory cytokines in the BEAS-2B cells. None of the phosphate or sodium concentrations tested altered *FGFR1* or *FGFR4* gene expression (Fig. 5a). Cells were pretreated with inhibitors of FGFR1-3 (AZD4547, iR1-3), FGFR4 (BLU-554, iR4), or both simultaneously before adding phosphate or sodium sulfate as a control. FGFR inhibition, independent of its isoforms, had no significant effect on IL6 or IL8 secretion into the conditioned media (Fig. 5b). Contrasting this, pretreatment of the bronchial epithelial cells with an inhibitor of ERK phosphorylation (U0126, iERK) before phosphate treatment reduced IL6 and IL8 protein levels markedly (Fig. 5c). The inhibitor effectively reduced ERK phosphorylation in both control and high phosphate treated BEAS-2B cells (Fig. 5d). Furthermore, pharmacological inhibition of sodium-dependent phosphate cotransporters with 5 mM PFA^42^ also significantly reduced phosphate-induced ERK phosphorylation (Fig. 5e). These data suggest that ERK activation induced by phosphate mediates proinflammatory cytokine secretion in BEAS-2Bs, which is independent of FGFR activation.

### Cigarette smoke exacerbates phosphate-induced cytokine secretion

**Figure 6:**
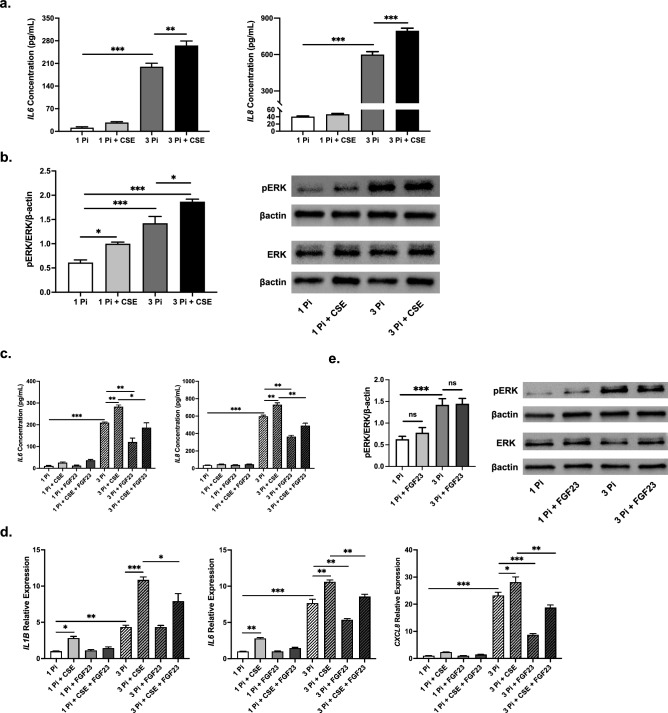
Cigarette smoke exacerbates phosphate-induced inflammatory cytokine expression and secretion, which is partially attenuated by FGF23. (**a**) IL6 and IL8 protein concentrations in conditioned media from BEAS-2B HBECs after 24 h sodium phosphate (Pi) stimulation with CSE. (**b**) Representative immunoblots and quantification of ERK phosphorylation in total protein extracts. βactin serves as loading control. (**c**) IL6 and IL8 protein levels in conditioned media after 24 h Pi stimulation with and without CSE and/or FGF23 cotreatments. (**d**) Relative mRNA transcript levels of *IL1B*, *IL6*, and *IL8* with Pi, CSE, and FGF23 cotreatments. (**e**) Representative immunoblots and quantification of ERK phosphorylation in total protein extracts from BEAS-2B HBECs after Pi stimulation with and without FGF23 cotreatment. βactin serves as loading control. All experiments were reproduced 3 times with triplicates. All values are mean ± SEM with *p ≤ 0.05, **p < 0.01, and ***p < 0.001. Original western blots presented are available in Supplemental Fig. S3. Statistical significance was analyzed via two-way (**a**,**b**,**e**) or three-way (**c**,**d**) ANOVA followed by Tukey’s multiple comparison post hoc test.

As smoking is one of the most common risk factors shared amongst patients with CKD and chronic lung disease^43^, we investigated the adverse synergistic effects of CS and phosphate on the BEAS-2Bs. Secreted IL6 and IL8 cytokine levels in the conditioned media were slightly higher, but not significant with cigarette smoke extract (CSE) alone. However, when given to cells in high phosphate conditions, the CSE induced significant increases in IL6 and IL8 secretion (Fig. 6a), which were significantly higher than treatment with high phosphate alone. ERK activation was increased after CSE treatment and exerted an additive effect when combined with 3 mM phosphate (Fig. 6b).

### FGF23 reduces phosphate and cigarette smoke-induced proinflammatory cytokine expression

Since high levels of serum phosphate in CKD patients are accompanied by elevated FGF23 to combat hyperphosphatemia, we added FGF23 treatments to our phosphate + CSE treatments to see whether their previously shown proinflammatory responses were further exaggerated. Surprisingly, IL6 and IL8 levels in the media were significantly reduced with the addition of FGF23, both with and without CSE in the 3 mM phosphate conditions (Fig. 6c). Similar trends were confirmed in the gene expression analysis using qRT-PCR of the BEAS-2B cells, with *IL1B*, *IL6*, and *CXCL8* mRNA levels all decreasing when FGF23 was added to the 3 mM phosphate and 3 mM phosphate + CSE treatments (Fig. 6d). Treatment of the cells with FGF23, together with either 1 mM phosphate or 3 mM phosphate, produced no significant changes in ERK phosphorylation (Fig. 6e) To assess whether alterations in cellular function, such as production of reactive oxygen species (ROS) or cell death, were contributing to activation of ERK and/or cytokine production, we evaluated H_2_O_2_ production and cell viability post-treatment. No significant differences were found between the groups tested in our experiments (Supplemental Fig. S4).

In summary, we show that elevated phosphate exacerbates CS-induced inflammation and ERK activation and that FGF23 can reverse this phosphate-mediated inflammatory cytokine secretion and gene expression in the bronchial epithelium. Furthermore, FGF23 also exerts this attenuating effect on the proinflammatory response exaggerated by the combination treatment with CSE and phosphate.

## Discussion

In this study, we show that dysregulated phosphate homeostasis, both as a result of genetic or adenine-induced CKD, induces airway inflammation and thickening of the bronchial epithelium. Our in vitro studies further examine the mechanism through which dysregulation of phosphate homeostasis affects the lungs, which indicate elevations of phosphate induce proinflammatory cytokine production in HBECs. Interestingly, FGF23 reduces proinflammatory cytokine production in both circumstances, indicating a protective effect of the hormone in the lungs during a hyperphosphatemic and/or diseased state.

Although this study is the first of our knowledge to characterize the bronchial epithelium and inflammatory lung environment of *Col4a3*^−/−^ (Fig. 1) and adenine-induced CKD mouse models (Fig. 2), various groups have previously described hyperphosphatemia as a potent contributor to inflammation in other organs^44–46^. Increased vascular calcification as a result of high serum and tissue phosphate concentrations have been shown to contribute to renal and cardiovascular inflammation in patients with CKD^44,45^. Additionally, excess dietary phosphate and adenine-induced CKD led to increased liver inflammation through stimulation of hepatic production of IL6 and IL1B in mice^8^. Supplying *Col4a3*^−/−^ mice with a low phosphate diet reduced functional iron deficiency and skeletal muscle wasting, factors that were exacerbated in the presence of hyperphosphatemia^8,46^. The evidence surrounding the link between excess phosphate and inflammation in multiple organs adds further credence to a mechanism supporting the pulmonary injury we observed in our murine models.

To our surprise, FGF23 alone had no effect on inflammatory gene expression or secretion in this cell type (Fig. 3), despite what we have observed in other HBECs in the past at similar concentrations. Our choice to treat the BEAS-2B HBECs with 40 ng/mL of FGF23 was based on previous work from our lab indicating that even 25 ng/mL induces significant secretion of IL1B in bronchial epithelial cells from COPD patients^16^. Additionally, 40 ng/mL has been utilized in our lab to evaluate inflammatory cytokine production in lung fibroblasts in combination with additional factors involved in phosphate homeostasis^38^. We cannot rule out that higher concentrations of FGF23 may have inflammatory effects in our cells, but our previous data suggests we would have expected to see at least some changes at the 40 ng/mL concentration used here. Although we saw no significant changes in ERK phosphorylation in lung homogenates from *Col4a3*^−/−^ or adenine-induced CKD mice, these homogenates included a mixture of all pulmonary cells, so we were unable to assess if specific differences were present in the bronchial epithelium in vivo.

We observed proinflammatory cytokine gene expression and secretion to be significantly higher in BEAS-2Bs exposed to a “high phosphate” environment, coupled with a robust activation of ERK (Fig. 4). AKT activation at Ser473 was unaffected, pointing towards a cell type-specific variance in the signaling that phosphate induces when referencing findings from studies examining adipocytes or endothelial cells^47,48^. Considering our results, it cannot be ruled out that phosphorylation of AKT at another site (Thr308 or Thr450) is not increased after the addition of phosphate. However, previous research indicates that hyperphosphatemia-induced phosphorylation of AKT at Ser473 in the lung without significant activation at the Thr308 site is sufficient to induce downstream signaling and subsequent tumorigenesis^49^.

We show that pharmacological inhibition of ERK phosphorylation can suppress half of the phosphate-induced IL6 secretion and two-thirds of the phosphate-induced IL8 secretion. Additionally, phosphate-induced ERK phosphorylation can be significantly blocked through inhibition of sodium-dependent phosphate cotransporters (Fig. 5). ERK activation and inflammation increased even further with the addition of CSE, exacerbating the existing proinflammatory environment caused by phosphate stimulation (Fig. 6). CS has been shown to cause these changes in previous studies examining HBECs^50,51^ and our results of an intensified effect in a high phosphate environment may potentially serve as one of several underlying signaling mechanisms that lead to increased obstructive lung disease susceptibility in CKD patients. Although smoking has been shown to be an independent risk factor for incident CKD, the mechanism has remained elusive. Our results indicate that ERK activation may be a player in the molecular signaling at play in these individuals, with CSE and elevated phosphate levels costimulating the same pathway.

The role FGF23 plays as a main regulator of phosphate homeostasis may provide some insight as to why we observe a protective effect with its addition in our study. However, the mechanism is still unclear and should be further examined. In contrast, our previous studies indicated a proinflammatory effect in the COPD bronchial epithelium, which could have been due to epigenetic changes of the bronchial epithelial cells themselves, leading to the opposite outcome^16^. Though, another of our previous studies showed a similar beneficial effect of FGF23 in pulmonary fibroblasts^38^ and points to the differential effects of FGF23 based on the context and environment. In this study, the addition of FGF23 to cells treated with high phosphate, with and without the addition of CSE, reduced proinflammatory cytokine expression and secretion in BEAS-2Bs. Previous reports have found that FGF23 stimulates ERK phosphorylation in the proximal tubules of the kidney^2^, but we found no such changes in the HBECs used in this study (Fig. 6). As alluded to earlier, we have shown that FGF23 can contribute to activation of mothers against decapentaplegic homolog 3 (SMAD3) activation^38^, which can contribute anti-inflammatory effects in the lung^52,53^, and can work in tandem with other phosphate homeostasis molecules to block cytokine production induced by transforming growth factor beta (TGFB)^16^. Other groups have also reported that FGF23 can play a role in the impairment of neutrophil recruitment and host defense during CKD^54^, which may provide some indication of why we saw decreases in inflammatory cytokine levels after the addition of FGF23 in our study. Nevertheless, further investigation of tissue- and disease-specific effects of FGF23 should be further studied to more thoroughly define its immune function in the body.

Our study has a few limitations. Unfortunately, both the *Col4a3*^−/−^ and adenine-induced CKD mice used in this study are too sick to introduce accompanying acute or chronic lung inflammation. Ideally, assessing lung injury post-chronic cigarette smoke exposure to model COPD^55^ would give a clearer picture of in vivo kidney-lung crosstalk and lung disease susceptibility accompanying hyperphosphatemia. Nevertheless, existing lung inflammation present in these two murine models suggests that additional lung injury may be exacerbated by further stimuli. Furthermore, although we identify some key regulators of phosphate signaling in the lung, the specific sensor that potentiates the actions of phosphate in this hyperphosphatemic environment remains elusive and should be further evaluated in future studies.

**Figure 7:**
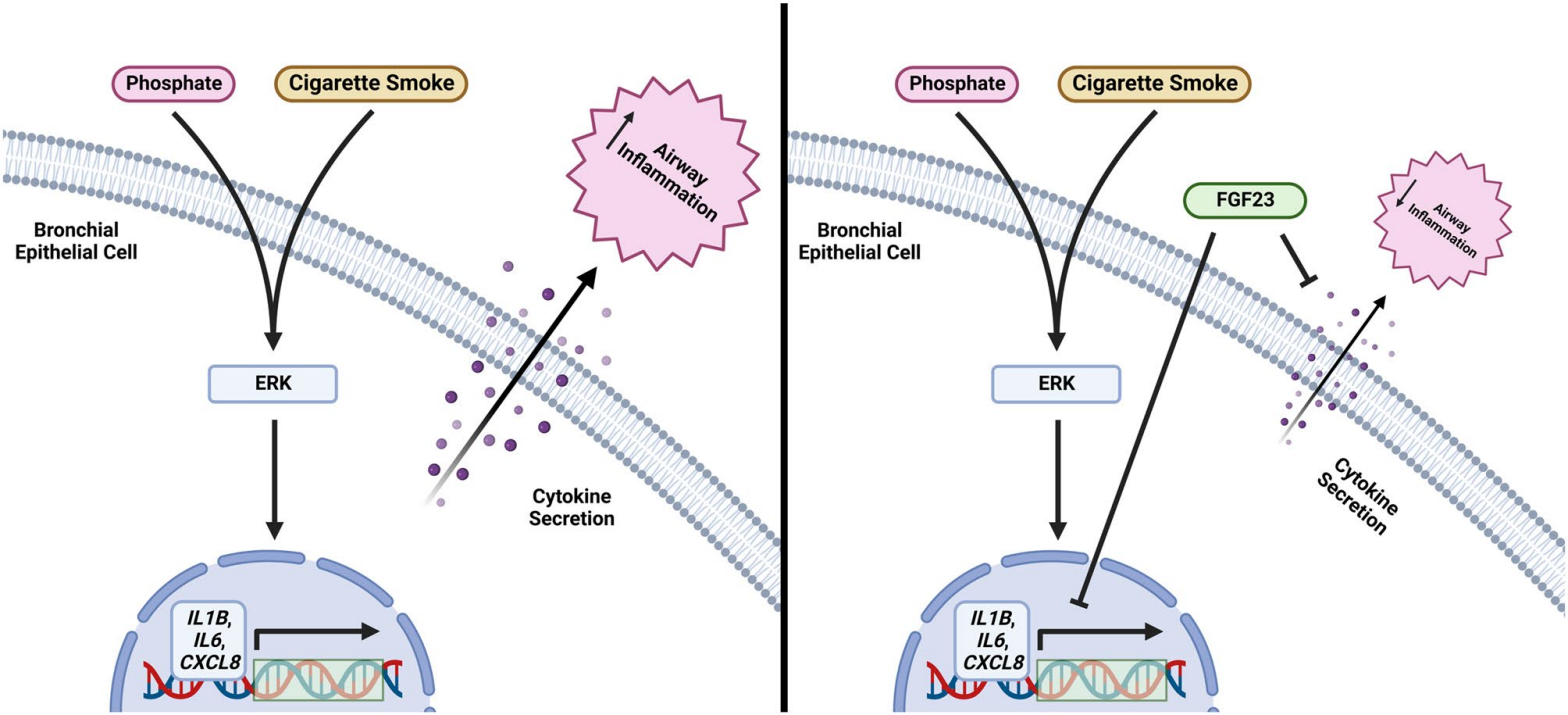
Schematic of the effects of high phosphate on proinflammatory cytokine expression and secretion in the presence of cigarette smoke/FGF23 in the bronchial epithelium.

In summary, we found that disruptions in phosphate homeostasis contributes to pulmonary inflammation and that high phosphate conditions make bronchial epithelial cells more susceptible to additional inflammatory stimuli. We established that hyperphosphatemia, as found in CKD, stimulates proinflammatory cytokine expression and secretion in HBECs through ERK activation. This inflammation is exacerbated in the presence of additional stimuli, such as CSE, suggesting a combinatorial effect from phosphate and CSE that may provide insight into the susceptibility and worsened pulmonary outcomes that CKD patients have involving obstructive lung disease and/or CS (Fig. 7). Currently, hyperphosphatemia in CKD patients is commonly managed with oral phosphate binders, but it is unclear whether they improve clinical outcomes in these patients^56^. Clinical studies are ongoing to investigate the efficacy of phosphate transporter inhibitors^57^ in combating hyperphosphatemia in CKD patients, but these are still very preliminary. Our study provides key insight into the link between CKD and susceptibility to obstructive lung disease and provides a therapeutic target that can be utilized in the development of treatments for patients who fit these criteria. Based on this, evaluating treatment plans for existing CKD patients at risk of developing lung disease and designing future studies to assess the pulmonary system in individuals with hyperphosphatemia is needed.

## Supplementary Information

Supplementary Information.

## Data Availability

The data generated or analyzed during this study are available from the corresponding author upon reasonable request.
