# Supplementary material for: Phosphate induces inflammation and exacerbates injury from cigarette smoke in the bronchial epithelium
Source: Sci Rep. 2023 Mar 25;13:4898. doi: 10.1038/s41598-023-32053-1 (PMC10039898; doi:10.1038/s41598-023-32053-1)
Supplement: Supplementary file 1 — Supplementary Information. [file 41598_2023_32053_MOESM1_ESM.pdf]

Phosphate induces inflammation and exacerbates injury from cigarette smoke in the bronchial epithelium

Seth Bollenbecker, Kylie Heitman, Brian Czaya, Molly Easter, Meghan June Hirsch, Shia Vang, Elex Harris, E. Scott Helton, Jarrod W. Barnes, Christian Faul, Stefanie Krick

Supplementary Figures

Supplementary Figure S1a:

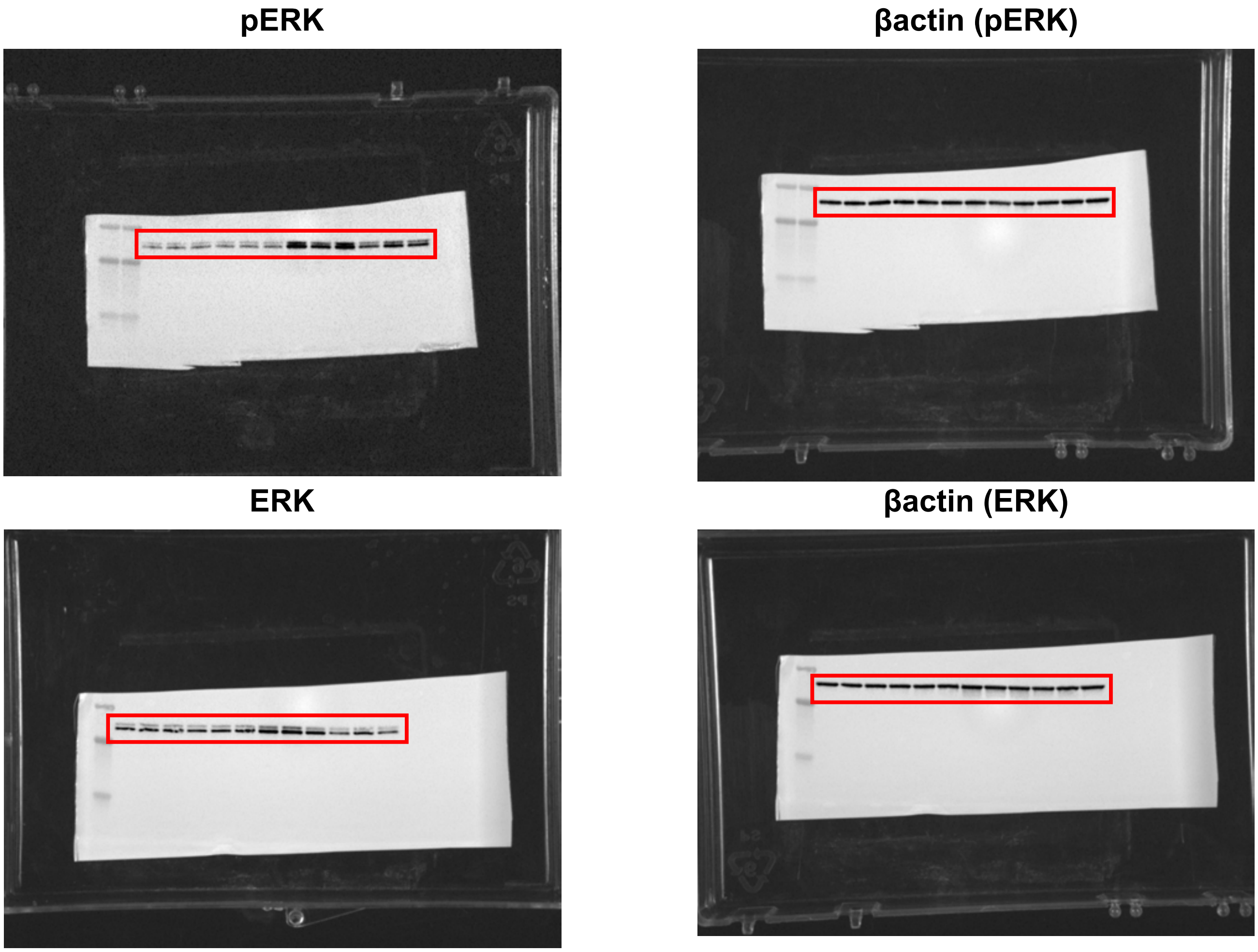

Supplementary Figure S1a: Original western blot images shown. For the manuscript, a horizontal region was cropped for the bands of interest (indicated by a red box on each picture). No additional modification was done. The images were then placed into Figure 4c.

Supplementary Figure S1b:

pAKT

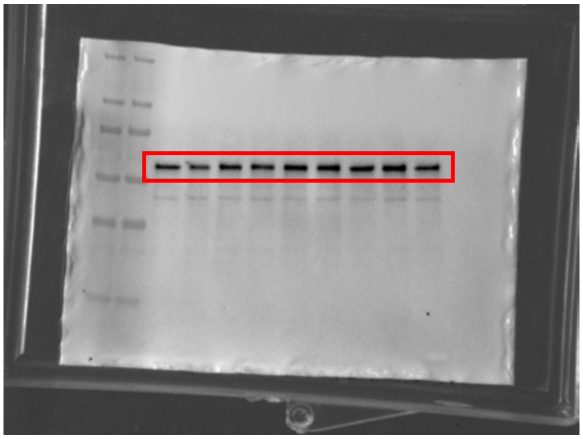

βactin (pAKT)

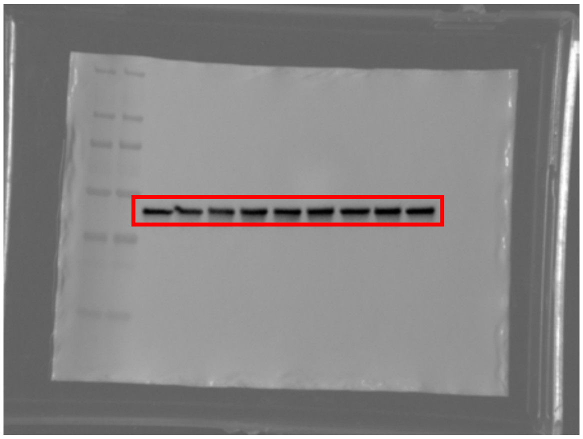

AKT

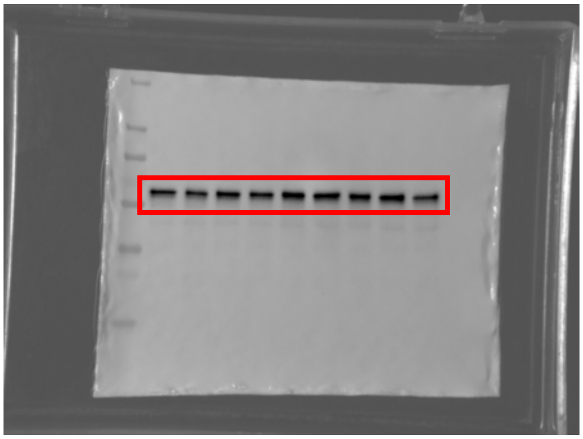

βactin (AKT)

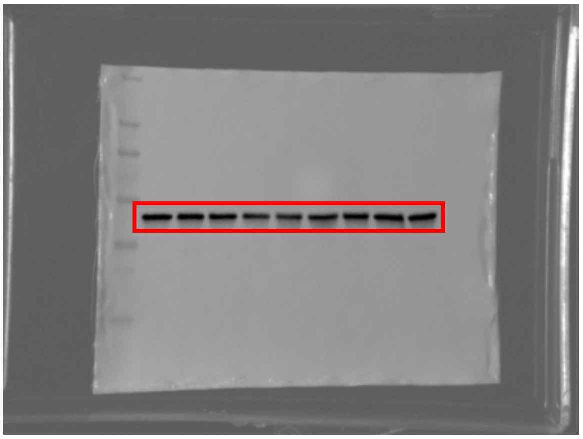

Supplementary Figure S1b: Original western blot images shown. For the manuscript, a horizontal region was cropped for the bands of interest (indicated by a red box on each picture). No additional modification was done. The images were then placed into Figure 4d.

Supplementary Figure S2a:

pERK

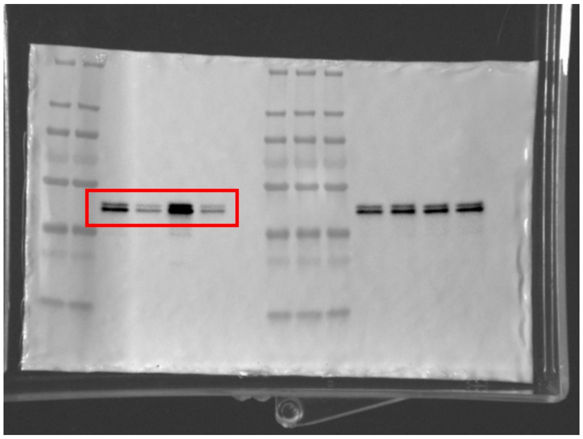

βactin (pERK)

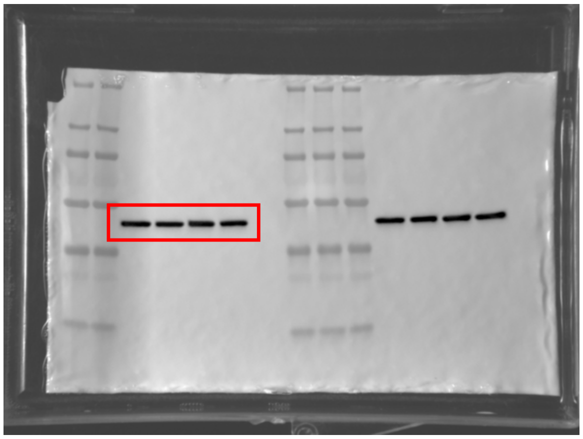

ERK

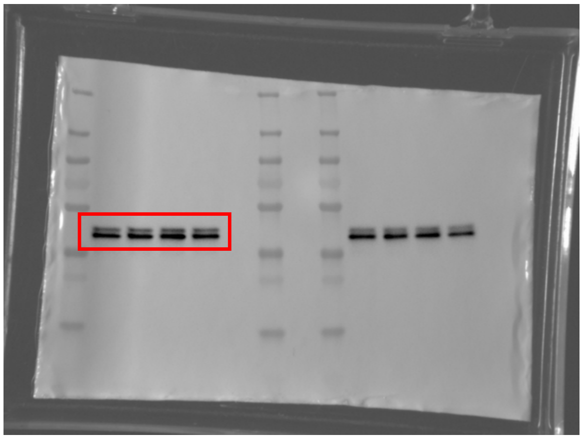

βactin (ERK)

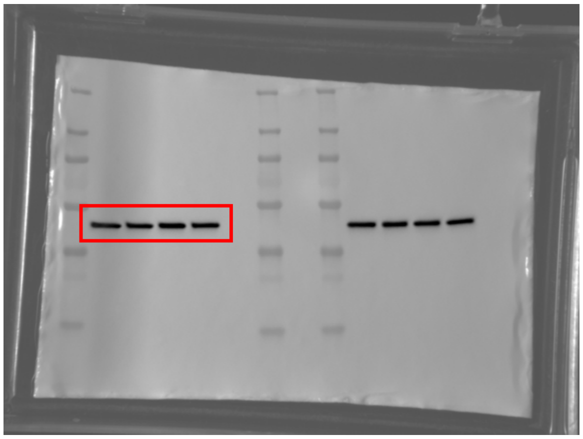

Supplementary Figure S2a: Original western blot images shown. For the manuscript, a horizontal region of the four bands on the left was cropped (indicated by a red box on each picture). The four bands on the right represent data for an unrelated experiment and were not included. The images were then placed into Figure 5d.

Supplementary Figure S2b:

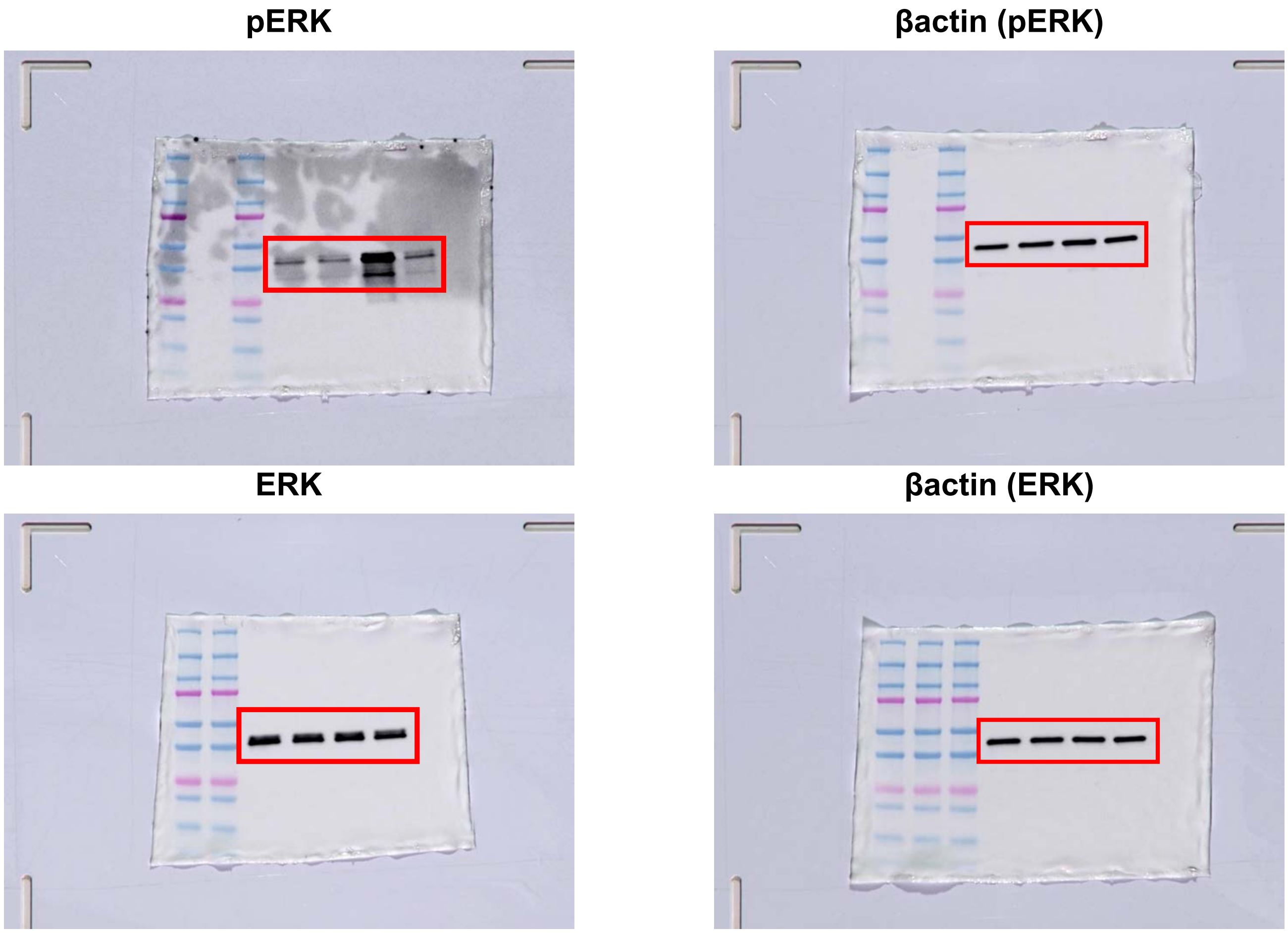

Supplementary Figure S2b: Original western blot images shown. For the manuscript, a horizontal region was cropped for the bands of interest (indicated by a red box on each picture). Cropped images from this figure in the manuscript were converted to grayscale to properly match the rest of the western blots taken on an alternate imager with no color camera. The images were then placed into Figure 5e.

Supplementary Figure S3a:

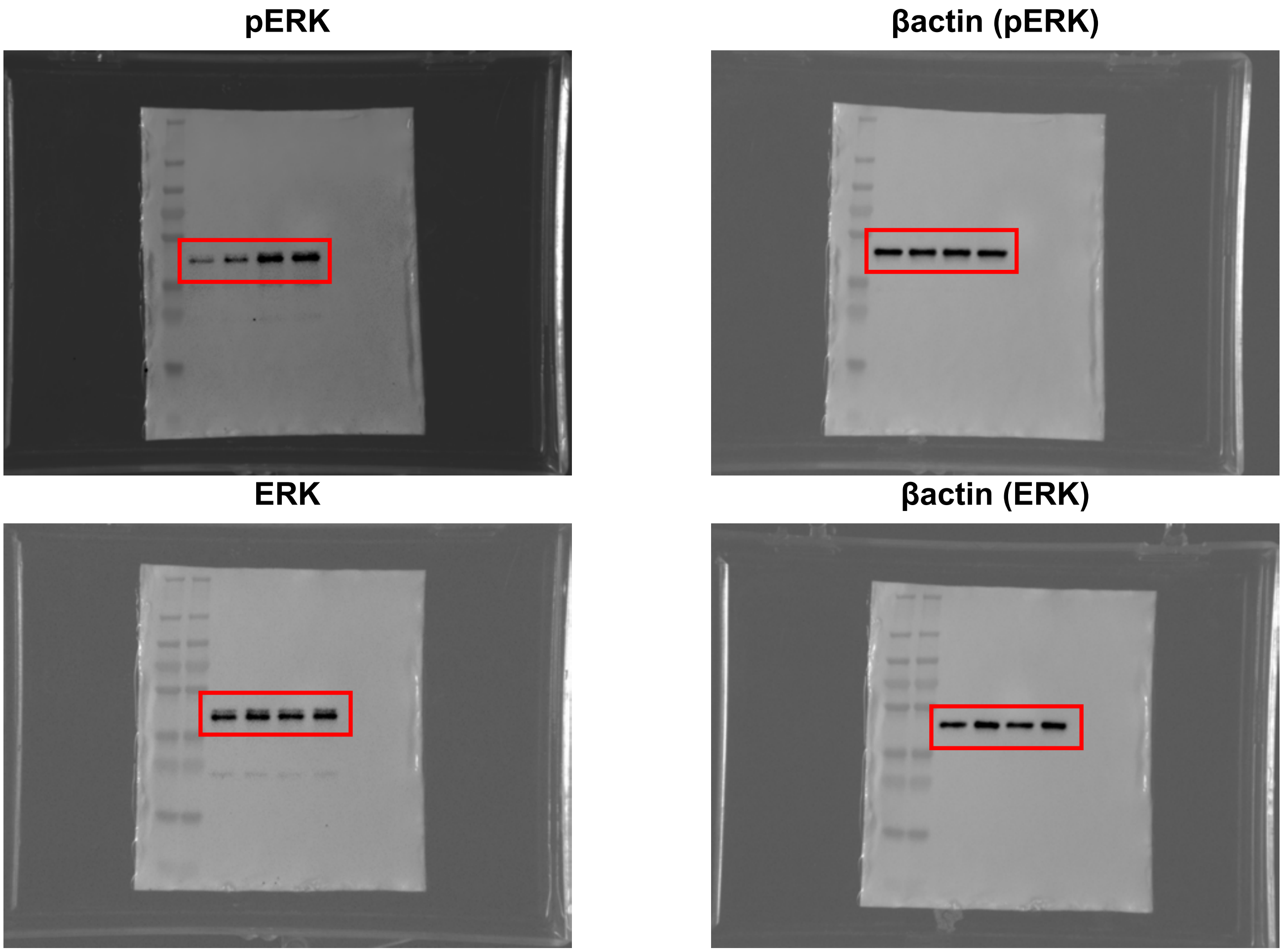

Supplementary Figure S3a: Original western blot images shown. For the manuscript, a horizontal region was cropped for the bands of interest (indicated by a red box on each picture). No additional modification was done. The images were then placed into Figure 6b.

Supplementary Figure S3b:

pERK

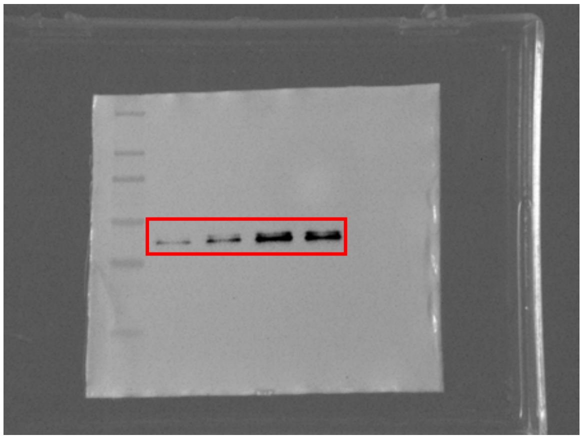

βactin (pERK)

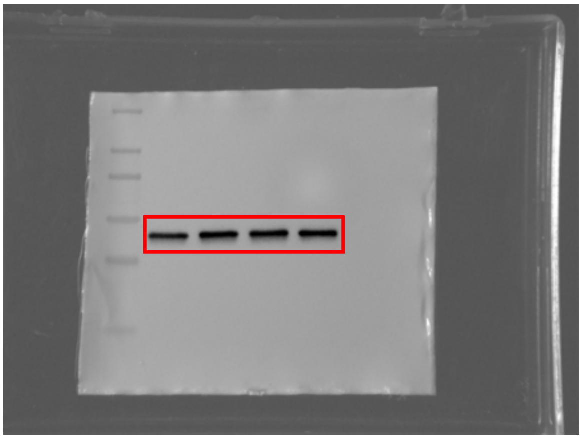

ERK

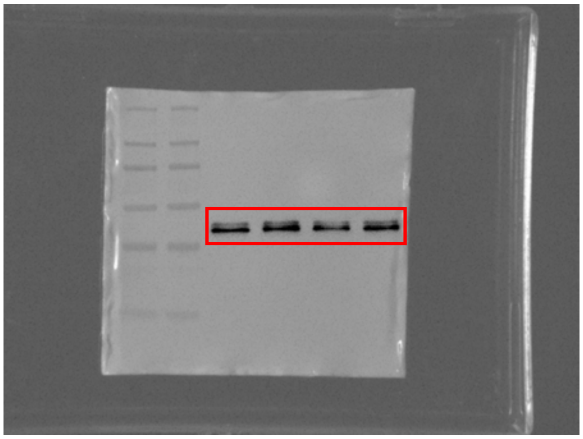

βactin (ERK)

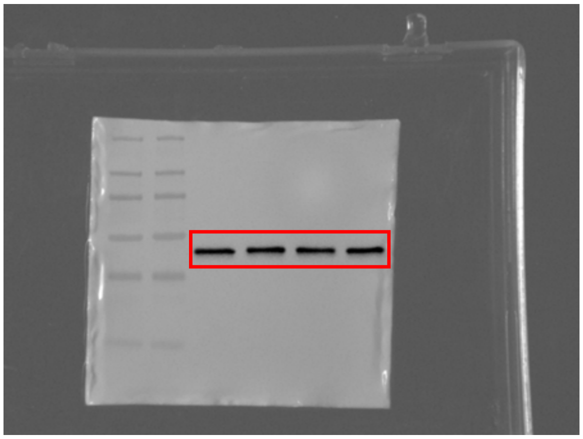

Supplementary Figure S3b: Original western blot images shown. For the manuscript, a horizontal region was cropped for the bands of interest (indicated by a red box on each picture). No additional modification was done. The images were then placed into Figure 6e.

Supplementary Figure S4a:

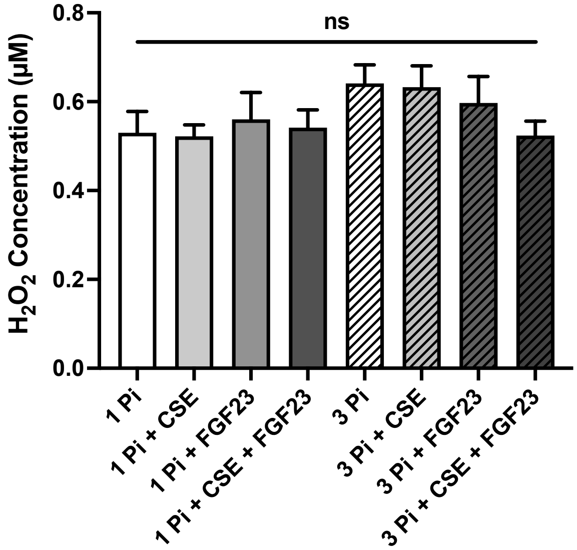

Supplementary Figure S4a: H_2O_2 concentration in BEAS-2B HBECs after 24-hour exposure to treatments listed for each group. Statistical significance was analyzed via three-way ANOVA with no significance found between any of the groups.

Supplementary Figure S4b:

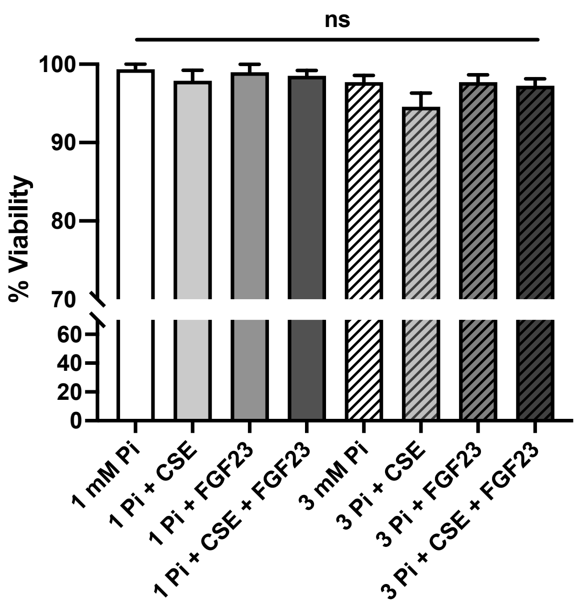

Supplementary Figure S4b: Percent viable BEAS-2B HBEC cells present after 24-hour exposure to treatments listed for each group, as measured by trypan blue exclusion test of cell viability. Statistical significance was analyzed via three-way ANOVA with no significance found between any of the groups.
